# Cross-Modal Stimulus Conflict: The Behavioral Effects of Stimulus Input Timing in a Visual-Auditory Stroop Task

**DOI:** 10.1371/journal.pone.0062802

**Published:** 2013-04-29

**Authors:** Sarah E. Donohue, Lawrence G. Appelbaum, Christina J. Park, Kenneth C. Roberts, Marty G. Woldorff

**Affiliations:** 1 Center for Cognitive Neuroscience, Duke University, Durham, North Carolina, United States of America; 2 Department of Neurobiology, Duke University, Durham, North Carolina, United States of America; 3 Department of Neurology, Otto-van-Guericke University Magdeburg, Magdeburg, Germany; 4 Leibniz Institute for Neurobiology, Magdeburg, Germany; 5 Smith College, Northampton, Massachusetts, United States of America; 6 Department of Psychiatry and Behavioral Sciences, Duke University, Durham, North Carolina, United States of America; 7 Department of Psychology and Neuroscience, Duke University, Durham, North Carolina, United States of America; Université catholique de Louvain, Belgium

## Abstract

Cross-modal processing depends strongly on the compatibility between different sensory inputs, the relative timing of their arrival to brain processing components, and on how attention is allocated. In this behavioral study, we employed a cross-modal audio-visual Stroop task in which we manipulated the within-trial stimulus-onset-asynchronies (SOAs) of the stimulus-component inputs, the grouping of the SOAs (blocked vs. random), the attended modality (auditory or visual), and the congruency of the Stroop color-word stimuli (congruent, incongruent, neutral) to assess how these factors interact within a multisensory context. One main result was that visual distractors produced larger incongruency effects on auditory targets than vice versa. Moreover, as revealed by both overall shorter response times (RTs) and relative shifts in the psychometric incongruency-effect functions, visual-information processing was faster and produced stronger and longer-lasting incongruency effects than did auditory. When attending to either modality, stimulus incongruency from the other modality interacted with SOA, yielding larger effects when the irrelevant distractor occurred prior to the attended target, but no interaction with SOA grouping. Finally, relative to neutral-stimuli, and across the wide range of the SOAs employed, congruency led to substantially more behavioral facilitation than did incongruency to interference, in contrast to findings that within-modality stimulus-compatibility effects tend to be more evenly split between facilitation and interference. In sum, the present findings reveal several key characteristics of how we process the stimulus compatibility of cross-modal sensory inputs, reflecting stimulus processing patterns that are critical for successfully navigating our complex multisensory world.

## Introduction

Events in the world often stimulate more than one sensory system. Under normal circumstances, an event will produce corresponding information that, although it enters through different sensory organs, is seamlessly integrated into a multisensory object when appropriate, while events that arise from differing sources in space and time are appropriately segregated. Multisensory interactions (e.g., whether cross-modal inputs are integrated or segregated), however, can be altered by variation in the relative compatibility between the different sensory inputs, the relative timing of their arrival, and how attention is allocated to the scene. Manipulations to these three basic cross-modal relationships can provide powerful techniques by which to tease apart the mechanisms underlying effective human information processing and cognitive control.

Stimulus compatibility effects arise when different sensory stimuli that occur nearby in time (either within the same modality or between different modalities) would tend to lead to different behavioral outcomes. Specifically, costs and benefits in performance can occur whenever differing dimensions of stimulus input have a sufficiently high degree of perceptual or semantic overlap such that they rely on common processing mechanisms. The color-naming Stroop task [1], in particular, has a long history of use in the visual domain for investigating information processing under situations of differing stimulus compatibility and/or levels of conflict. In its traditional version, this task has participants name the color of the ink or font that a word is written in, while ignoring the meaning of the word. The relevant dimension (the ink color) and the irrelevant one (the word meaning) can be congruent and match (“Blue” written in blue ink), or can be incongruent and signal different responses (“Blue” written in red ink), the latter leading to behavioral costs, including slower response times (RTs) and reduced accuracy (see [2] for review). Many studies of the Stroop task (e.g., [3]) often also include a neutral condition of some sort, wherein the meaning of the word does not match up with any of the potential responses (“Blue” written in green ink, where the color green was not one of the response options). Comparisons between the responses to these various conditions can provide useful measures of both facilitation (improved processing for congruent relative to neutral) and interference (impaired processing for incongruent relative to neutral), as well as yielding measures of the ‘Total Stroop Effect’ (differences between incongruent and congruent; [4]).

The degree to which cross-modal conflict patterns are similar to within-modality conflict remains a much less explored question. Evidence from behavioral [5], [6], [7], [8] and neural studies [9], [10] of cross-modal conflict have shown that stimulus incompatibility across the different modalities can produce conflict effects in a similar manner to those observed in unimodal visual-conflict tasks (e.g., [11]; but see [12]). Two common patterns have emerged from this literature: first, in cases where complex stimuli are involved (e.g., letters or pictures and words), visual processing tends to be overall faster than auditory processing as reflected by RTs [9], [10]. Second, there is a general pattern of asymmetry between the magnitudes of conflict effects observed in cross-modal contexts, in that task-irrelevant incongruent visual stimuli generally produce more interference on the processing of behaviorally relevant auditory stimuli, than vice versa [9], [10], [13]; but see [5]. Such cross-modal asymmetries are often found in environmental instances of uncertainty wherein the more reliable modality ‘wins’ (e.g., a shift in the auditory percept toward a more spatially reliable visual stimulus, as in the ventriloquist illusion [14], [15]). Indeed, when Yuval-Greenberg and Doeuell degraded their visual stimuli making them more difficulty to identify, the visual conflict effect on auditory processing diminished substantially, suggesting that the visual information was not being weighted as heavily [10]. These previous findings, however, concerning the relative speeds of processing for auditory and visual stimuli, as well as the asymmetric levels of interference between the two modalities (visual larger than auditory), have only been reported from circumstances when auditory and visual stimuli were presented simultaneously, and thus they have not been mapped out as a function of the relative onsets of the audio-visual stimuli. The variation of such onsets seem likely to ramify in both speed and strength-of-processing effects of these cross-modal interactions, which could help provide insight into the underlying mechanisms of multisensory processing.

Indeed, in the visual modality one approach that has yielded insight into the facilitation and interference effects resulting from stimulus incompatibility has been to vary the timing between the relevant and irrelevant stimulus features (e.g., [16], [17], [18], [19], [20], [21], [22]). Such stimulus onset asynchrony (SOA) manipulations make it possible to map the full time course of incongruency interactions between stimulus components, thereby helping to inform cognitive models as to the underlying processes that enable the executive function processes that are invoked to address such conflict. Using this technique, previous within-vision work from our group [23], [24] and others [25] have found that the occurrence of task irrelevant distractors prior to the relevant target has the capacity to enhance the magnitude of incongruency effects. Similarly, this work has also shown that the post-exposure of irrelevant distractors is still able to influence the behavioral responses to the relevant target even when it presented up to 200 ms after the target stimulus. Using this SOA manipulation in a cross-modal context can provide valuable information about the strength and timing of interactions that arise through audio-visual stimulation.

The current study sought to fully characterize the effects, interactions, and time course of cross-modal incongruency as revealed in the behavioral responses in an auditory-visual SOA-varying Stroop task. To this end, we had participants attend to color-word stimuli in either the auditory modality or the visual modality while being presented with congruent, incongruent, or neutral stimuli in the other (irrelevant) modality. The relative onsets of the relevant/irrelevant stimuli were varied from +400 ms to –400 ms in increments of 100 ms to map the time course of behavioral incongruency interactions, with additional unimodal trial types being included as controls to provide a baseline reference. In that previous work in our lab using visual Stroop stimuli [24] has found different patterns of incongruency effects when the SOAs were presented randomly versus being blocked together in the same experimental run, this factor (‘SOA-arrangement’) was also included in the present design. This multifactorial design thus allowed us to delineate the relative strengths and timing of processing interactions as they relate to congruent and incongruent cross-modal inputs. Moreover, we employed psychometric modeling techniques to derive the time course of the interference effects and more fully estimate the relative speed of cross-modal stimulus interactions. Collectively, the present experimental design allows for the replication and extension of previous reports of cross-modal dominance effects (e.g., [26], [27]) and temporal manipulations of audio-visual stimulus input (e.g., [7], [28], [29]). By combining these various factors together we aimed to derive a comprehensive picture of cross-modal conflict processing and to determine how these effects increase and decrease within and beyond the temporal window of integration.

## Methods

### Ethics Statement

All of the methods and procedures described below were approved by the Institutional Review Board at Duke University. All participants gave written informed consent and were compensated for their time at a rate of $15/hour.

### Participants

Fifteen healthy, right-handed volunteers are included in the final analysis for this two-session study (mean age = 22.9 years, 6 female). Three additional participants were excluded due to failure to return for the second session of testing, and five additional participants were excluded due to a poor percentage of total responses (i.e., having at least one session for which, on over half the trial types, their proportion of responses was less than 2 standard deviations from the mean proportion of responses across subjects). All participants were native English speakers with normal visual acuity and normal color vision.

### Experimental Design and Procedure

**Figure 1:**
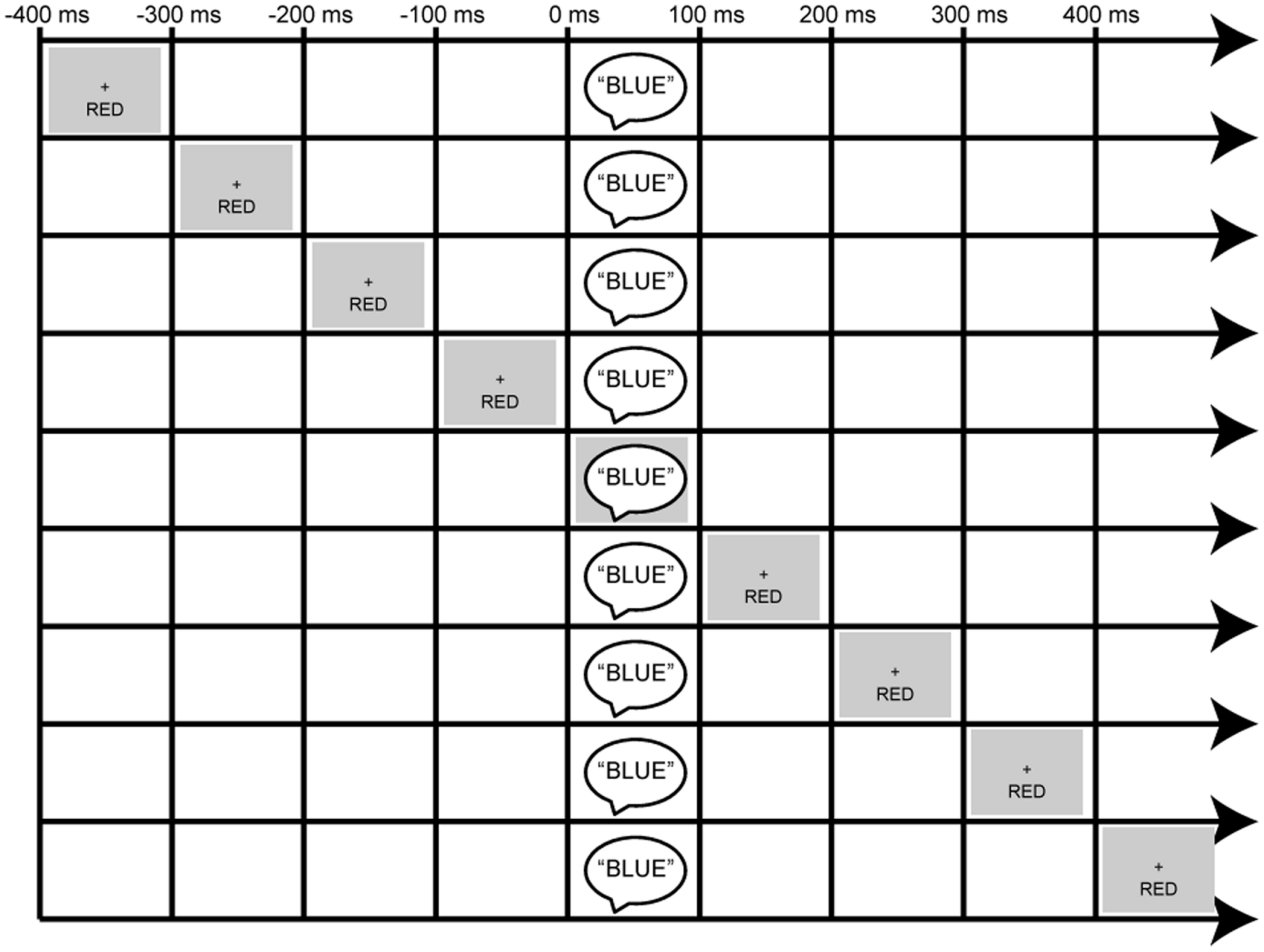
Schematic of Experimental Design. Example shown is that of an incongruent trial for the auditory attention condition wherein participants were instructed to report the auditory stimulus component (spoken-word “BLUE”) while ignoring the visual stimulus component (in this case the word “RED”, presented visually below fixation). The irrelevant visual information could come before or after the target in increments of 100 ms out to −400 and +400 ms.

Experimental stimuli consisted of auditory spoken words and visual typeset words (see Figure 1 for task design). Auditory stimuli were the spoken words “Red”, “Blue”, “Green”, “Yellow”, “Pink”, “Brown”, and “Orange”. These words were recorded from a male, native-English speaker and had an average duration of 385 ms with 20 ms rise time and a 20 ms fall time. The auditory stimuli were presented at 50 dB (SPL) centrally through two speakers positioned to the left and right of the CRT monitor with a 60 Hz refresh rate. Visual stimuli were the corresponding written words “RED”, “BLUE”, “GREEN”, “YELLOW”, “PINK”, “BROWN”, and “ORANGE” printed in black Arial font on a gray background. The center of the words was 3.75° below fixation, and participants were seated 57 cm from a CRT monitor. The visual stimuli were presented for 385 ms. A central white fixation cross remained on the screen for the duration of the experiment. All stimuli were presented via Presentation (Neurobehavioral Systems).

The experimental design consisted of four independent variables that were varied within subject – namely ‘*Incongruency’*, *Stimulus Onset Asynchrony (SOA)*, ‘*SOA-arrangement*’, and *‘Attended Modality*’ – along with a fifth variable *‘Response Button Mapping’* that was varied between subjects.

The first independent variable ‘*Incongruency’* was defined by the correspondence between the color of the spoken and written words on each trial. In all experimental sessions the auditory and visual stimulus components were arranged in three equally frequent configurations that comprised congruent, incongruent, and neutral pairings, presented in randomized order. In a congruent trial type, the auditory and visual stimuli matched (e.g., the spoken word “Red” was paired with the visual word “RED” printed on the screen). The incongruent trial types consisted of auditory and visual stimuli that did not match, but for which there was a specific assigned response mapping for the non-corresponding incongruent stimulus component (e.g., the spoken word “Red” paired with the visual word “BLUE”). The neutral trials consisted of auditory and visual stimuli that did not match, but for which the irrelevant stimulus component was not mapped to one of the 4 response buttons (e.g., the spoken word “Red” paired with the visual word “BROWN,” where BROWN was not a response option). Additionally, unimodal-auditory and unimodal-visual stimulus trials in which the target, spoken-word or written-word was not accompanied by any cross-modal distractor were also presented within the randomized sequence.

The second independent variable ‘*SOA’* reflected the asynchrony between the presentation of the task-irrelevant distractor word and the target word. There were nine levels of SOA; −400, −300, −200, −100, 0, +100, +200, +300, and +400 ms, so that the task-irrelevant stimulus component could precede the target, occur simultaneously with it, or follow it. A total of 36 trials were presented for each SOA and incongruency condition (e.g., −400 ms SOA, congruent pairing). The trial onset asynchrony (i.e., the time from the start of one trial to the start of the next) was jittered randomly between 1600–1800 ms to prevent temporal predictability, particularly in the case of the blocked version of the ‘SOA-Arrangement’ variable (see below).

The third independent variable was the ‘*Attended Modality.’* During half of each experimental session participants were instructed to attend to the auditory modality and report the identity of the auditory word with a button press while ignoring the visual stimuli. On the other half of the trials participants were instructed to attend to the visual modality, report the written word with a button press, and to ignore the auditory stimuli. The order of the attended modality was randomized and counterbalanced across participants. Across both attended modalities, the relevant stimuli (i.e., those to which a response was mapped) were the words “Red”, “Green”, “Blue”, and “Yellow.”

The fourth independent variable was ‘*SOA-Arrangement.’* Previous work from our lab [24] has found that within the visual modality the arrangement of the SOAs within a run can affect the temporal patterns of facilitation and interference (see Discussion for details). To determine if this also occurs with cross-modal stimuli, we manipulated the arrangement of the different SOA conditions across experimental blocks. In two separate experimental sessions, collected on separate days, participants were either presented with a ‘*random-SOA*’ arrangement in which all nine SOA conditions were intermixed and appeared randomly within each experimental block, or they were presented with a ‘*constant-SOA’* arrangement in which the same SOA was presented on every trial in an experimental block. The order of the constant-SOA and random-SOA session was counterbalanced over subjects. For the constant-SOA-arrangement session, the order of the specific SOA blocks was also randomized across participants.

The fifth independent variable was the ‘*Response Button Mapping.’* To control for any confounds with the specific target colors being mapped to specific buttons, we used two different response mappings in these experiments. For half of the participants the target words “Red”, “Green”, “Blue” and “Yellow” were mapped to the “D”,”F”, “J”, and “K” keys respectively. For the other half of the participants this mapping was flipped (left-to-right hand and index-to-middle finger) such that the mappings were to the “J”, “K”, “D”, and “F” key respectively. Participants always utilized both hands to respond, with their index and middle fingers positioned on the keyboard as if they were typing. Planned analyses revealed that behavioral performance did not differ as a function of these two button-assignment mappings (there was no main effect of response button mapping on accuracy (*F<*1) or on response time (<1), and the response button mapping did not significantly interact with any of the other factors in the accuracy (all *p*’s>0.05) and in response-time analysis (all *p*’s>0.05). Subsequent analyses reported below were therefore collapsed over the factor of *Response Button Mapping*.

For all tasks, participants were instructed to maintain central fixation and were monitored through a closed-loop video camera to assure that they were consistently looking at the fixation cross and maintaining the proper distance from the screen. Each run consisted of 108 trials and lasted approximately 3 minutes. A total of 20 runs were collected for each participant (10 in each experimental session), and participants were given the opportunity to rest between the runs. Prior to the start of each experimental session, participants were given a short block where they practiced the response mappings to unimodal stimuli in that modality, followed by a second practice block wherein the distractors from the other modality were present. Performance was monitored during these blocks to ensure that the task was being performed correctly.

### Behavioral Analysis and Modeling

Trials were counted as correct if the responses occurred between 200 and 1200 ms following the presentation of the target (attended) stimulus component. This excluded ∼2% of correct responses that fell outside of this range. Data were collapsed over the different colors and response mappings to arrive at within-participant mean response times (RTs; correct trials only) and accuracy measures for the various levels of the other factors. RT and accuracy data were submitted to separate 4-way repeated-measures analyses of variance (rANOVA), with factors SOA-Arrangement (2-levels; blocked and random), Attended Modality (2-levels; visual and auditory), SOA (9 levels), and Incongruency (3 levels; congruent, neutral, incongruent). Additional two-tailed, paired t-tests were performed on specific comparisons to more precisely delineate effects are described in more detail in the appropriate Results sections below. The significance thresholds were set to a p-value of 0.05 and, when applicable, adjusted using the Greenhouse-Geisser correction for non-sphericity or Bonferroni correction for multiple comparisons.

In order to ensure that any effects of incongruency we were observing were not due to the fact that one modality had overall slower RTs than the other (auditory being slower than visual), we conducted an additional analyses on the data following a modality-normalization procedure. Using the RTs to the unimodal stimuli as a baseline, we took the RTs for each level of congruency and SOA and divided by the RT for the unimodal stimuli. For example the new value for the congruent RT in the −400 SOA of the ‘attend auditory’ condition would be equal to the original RT in that condition divided by the RT for the auditory alone. The normalized data were then entered into the same ANOVAs as described above.

A primary interest in this study was how these various conflict-related RT effects would change as a function of SOA. That is, how do the cross-modal interference effects decay over time as a function of attended modality? In order to try to characterize this relationship beyond what the ANOVAs could quantitatively describe, an incongruency-by-SOA psychometric curve fitting analysis was also performed. For this purpose, we adapted a curve-fitting procedure (modeled after [30]) that allowed the horizontal position along the x-axis (i.e., temporal position) of the incongruency by SOA function to be represented by a single horizontal-shift value and to be tested empirically. Specifically, we fit sigmoidal curves to each participant’s incongruency (I-C) by SOA reaction time function separately in the ‘attend auditory’ and ‘attend visual’ conditions. The sigmoidal function used had three free parameters that were optimized using a non-linear least-squares fitting algorithm in MATLAB (MATLAB Optimization Toolbox, Mathworks, Natick, MA, USA).
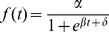



In the applied fitting function (see Eq. 1), α represents a vertical scaling of the sigmoid curve, β represents the steepness of the sigmoid curve, and δ indicates the translation of the curve along the x-axis (temporal shift). In order to constrain the fits within the vertical dimension, α was limited to be no greater than the maximum interference effect for a given subject in their visual and auditory conditions. From these fits we obtained the SOA at which the interference effect dropped by half (also represented by the point of inflection in the sigmoidal function), and then submitted these values to a paired t-test contrasting the attend-auditory and attend-visual conditions. The inflection point was chosen as a marker for the interference effects as it provided a principled point of comparison between each of the modalities as a result of the curve-fit functions. As no differences were observed in the ANOVA as a function of the SOA-arrangement and response-order mapping (see Results below), the data were collapsed across these factors for each subject prior to implementation of the curve fitting procedure.

## Results

### Accuracy

Accuracy was generally high across all tasks and conditions. A repeated-measures ANOVA with the factors of *SOA-Arrangement*, *Attended Modality*, *SOA arrangement*, and *Incongruency* was carried out to determine if task performance was modulated by any of these experimental manipulations. There was a main effect of incongruency on the accuracy (*F*(2,26) = 7.24, *p = *0.007, η*_p_^2^* = .36), with no other main or interactions (all p’s>.05). Posthoc specific comparisons showed that participants were more accurate at the Bonforroni-corrected alpha-level of 0.02 in the congruent (mean = 93.3%) and neutral (mean = 93.1%) conditions than in the incongruent (mean = 92.1%) condition (congruent vs. incongruent: *t*(14) = 2.87, *p = *0.01; neutral vs. incongruent: *t*(14) = 3.99, *p = *0.001), with accuracy between the congruent and neutral conditions not significantly differing (*t*(14) = 0.58, *p = *0.57). In sum, accuracy was only modulated by congruency, and, as such, the rest of the analysis focused on the response-time data.

### Response Times

**Figure 2:**
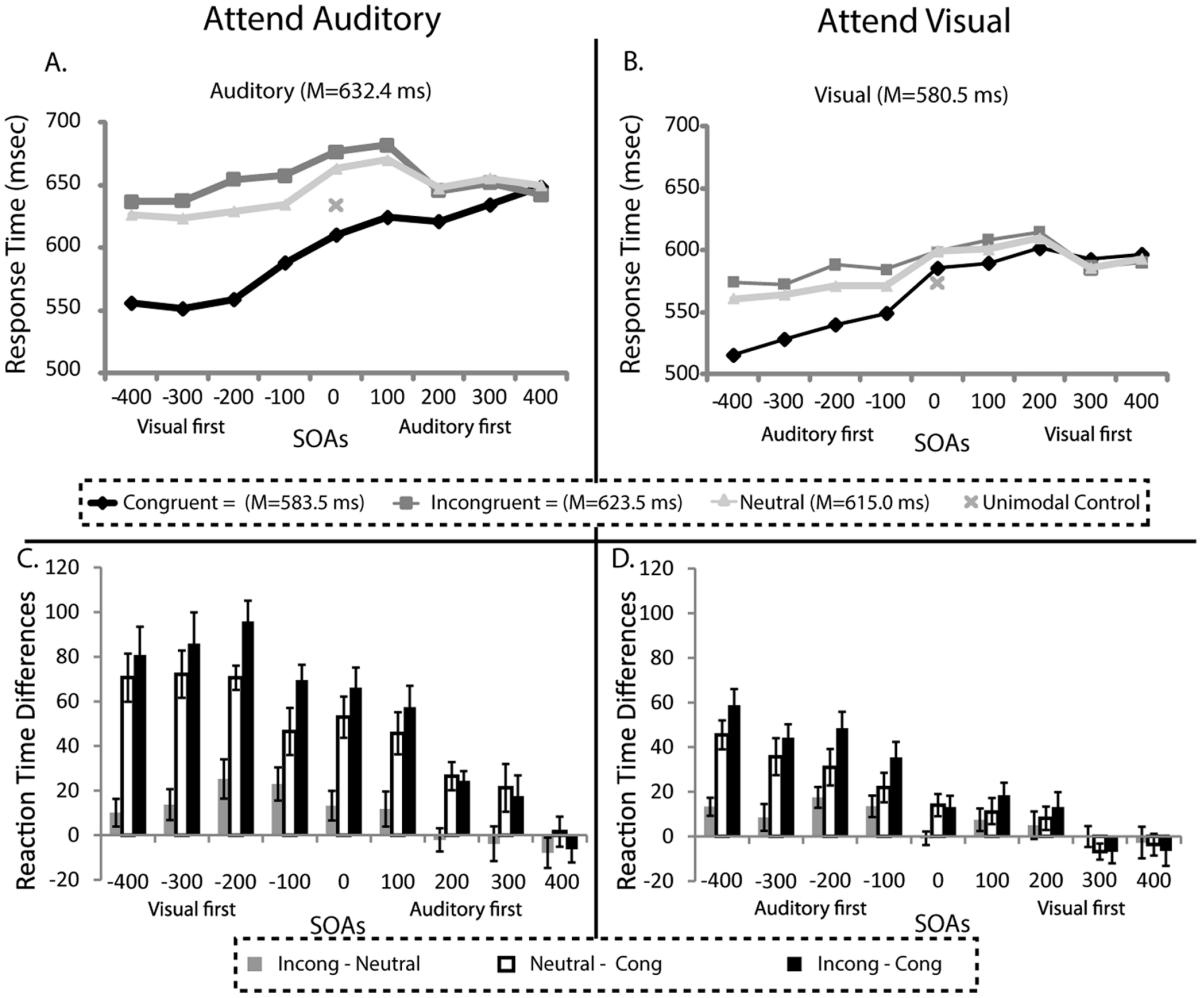
Response times. Top. Mean response times across participants (N = 15) plotted for the attend-auditory condition (A), and the attend-visual condition (B) by SOA, and congruency conditions. RTs were modulated by modality of attention (slower for auditory), SOA, and incongruency. X’s denote the mean RT for the unimodal auditory (left) and unimodal visual (right) stimuli, respectively. Bottom. The bottom panels show the RT differences between the congruency conditions as a function of SOA for the attend-auditory (C) and attend visual (D) conditions. Error bars denote the SEM. For the SOAs wherein the irrelevant information from the other modality came first there was a great amount of facilitation (neutral minus congruent) and full incongruency (incongruent minus congruent), with minimal interference (incongruent minus neutral). All of these effects tapered off as the irrelevant stimulus was presented later in time.

The primary focus of this study was to determine how the response times to cross-modal Stroop stimuli were modulated by the various design factors. To assess this, we first conducted repeated-measures ANOVA with the factors of *SOA-Arrangement*, *Attended Modality*, *SOA*, and *Incongruency,* with subjects as a repeated measure. This revealed a main effect of attended modality (*F*(1,14) = 19.4, *p = *0.001; η*_p_^2^* = .58), a main effect of SOA (*F*(8,112) = 30.8, *p*<0.001; η*_p_^2^* = .69), a main effect of incongruency (*F*(2,28) = 144.5, *p*<0.001; η*_p_^2^* = .91), an interaction of attended modality and incongruency (*F*(2,28) = 25.6, *p*<0.001; η*_p_^2^* = .65), and an interaction of SOA and incongruency (*F*(16,224) = 16.8, *p*<0.001; η*_p_^2^* = .55). There were no main effects of SOA-arrangement (*F*<0.1) and no significant interactions of this factor with any of the other factors (all *p*’s>0.1). Accordingly, all subsequent analyses were collapsed across the mixed-SOA and blocked-SOA trials. Figure 2 shows the response times plotted as a function of attended modality, SOA, and incongruency (top panel).

The main effect of attended modality was due to overall faster RTs in the attend-visual condition (*M* = 580.5 ms) compared to the attend-auditory condition (*M* = 632.4 ms). More importantly, however, was the interaction between attended modality and incongruency. To further assess what was driving this interaction, we took the differences shown above in Figure 2C and 2D between incongruent versus congruent (full incongruency), neutral versus congruent (facilitation) and neutral versus incongruent (interference), and then collapsed across SOA and compared as a function of attended modality. This revealed a significant difference for the full incongruency effect, wherein there was a greater incongruency RT difference when participants were attending to the auditory modality (*M = *55.5 ms) as compared to when they were attending to the visual modality (*M* = 24.4 ms; *t*(14) = 5.24, *p*<0.001 ). Further, there was significantly greater facilitation in the auditory attention condition (*M = *45.2 ms) than in the visual attention condition (*M = *17.6 ms; *t*(14) = 6.83, *p*<0.001). On the other hand, the relatively smaller interference effect did not differ across modalities (attend auditory *M* = 10.4 ms, attend visual *M = *6.8 ms, *p*>0.05).

Exploration of the main effect of SOA revealed that there was a significant linear trend across SOAs such that RTs increased as the irrelevant stimulus came later in time, regardless of its incongruency relationship (*F* (1,14) = 56.29; *p*<0.001), suggesting a possible general alerting effect of the irrelevant stimulus when it occurred first. This effect can be seen in Figure 2 (Top) where for both the auditory and visual attention conditions there is an overall positive slope in the RTs plots. In addition, to examine more specifically for the presence of such a general alerting effect that was a function of SOA and not due to incongruency *per se*, we conducted an ANOVA on the neutral condition, collapsed across modality, across the SOAs. This indeed revealed a significant linear trend (*F*(1,14) = 25.0, *p*<0.001; η*_p_^2^* = .64), with RTs increasing as the irrelevant neutral stimuli were presented later in time.

The main effect of incongruency was driven by significant differences between congruent (*M = *583.5 ms) and incongruent (*M = *623.5 ms, *t*(14) = 14.2, *p*<0.001), congruent and neutral (*M = *615.0 ms, *t*(14) = 11.3, *p*<0.001) and incongruent and neutral stimuli (*t*(14) = 6.88, *p*<0.001), as assessed with specific contrasts between these conditions. Therefore, the neutral condition was indeed different from both congruent and incongruent trial types, with the neutral response times falling between those of the other two trial types. Although these three conditions did differ from each other as a main effect, the magnitude of the differences varied as a function of SOA, with larger differences observed between neutral and congruent (facilitation) and incongruent and congruent (interference) when the irrelevant information came first (Figure 2, bottom). These qualitative differences emerged statistically in the significant interaction between SOA and incongruency, which were further explored by performing a series of post-hoc t-tests on the facilitation, interference, and full incongruency effects, collapsed across modality at each of the SOAs. Table 1 shows the results of these t-tests, revealing the presence of significant effects (Bonferroni corrected for 9 tests across the 9 SOAs) at both negative SOAs and at positive SOAs.

**Table 1:** T-tests examining the incongruency by SOA interaction for RTs, collapsed across attended modality and SOA-arrangement.

	−400	−300	−200	−100	0	100	200	300	400
Facilitation (Cong vs. Neut)	***t*** ** = 7.85, ** ***p*** **<0.001**	***t*** ** = 9.04, ** ***p*** **<0.001**	***t*** ** = 10.25, ** ***p*** **<0.001**	***t*** ** = 4.97, ** ***p*** **<0.001**	***t*** ** = 6.36, ** ***p*** **<0.001**	***t*** ** = 5.38, ** ***p*** **<0.001**	***t*** ** = 4.12, ** ***p*** ** = 0.001**	*t* = 1.24, *p* = 0.23	*t* = 0.23, *p* = 0.82
Interference (Incong vs. Neut	*t* = 2.70, *p* = 0.02	*t* = 2.39, *p* = 0.03	***t*** ** = 4.66, ** ***p*** **<0.001**	***t*** ** = 3.38, ** ***p*** ** = 0.004**	*t* = 1.64, *p* = 0.12	*t* = 1.81, *p* = 0.09	*t* = 0.43, *p* = 0.68	*t* = 0.49, *p* = 0.63	*t* = 1.02, *p* = 0.32
Full Interference (Incong vs. Cong)	***t*** ** = 8.05, ** ***p*** **<0.001**	***t*** ** = 9.09, ** ***p*** **<0.001**	***t*** ** = 11.47, ** ***p*** **<0.001**	***t*** ** = 11.13, ** ***p*** **<0.001**	***t*** ** = 7.72, ** ***p*** **<0.001**	***t*** ** = 7.47, ** ***p*** **<0.001**	***t*** ** = 5.02, ** ***p*** **<0.001**	*t* = 1.00, *p* = 0.33	*t* = 1.15, *p* = 0.27

Significant facilitation and full interference effects (at the Bonferroni corrected alpha level of 0.006 [corrected for 9 tests]) were found even when the irrelevant stimulus came 200 ms after the relevant one, noted with bold. The interference effects were generally smaller and lasted for substantially less time, only being significant up to −100 ms.

Finally, to determine how the RTs to the unimodal control stimuli compared to the RTs to the other trial types, we conducted planned comparisons between the RT of the unimodal control and the congruent, incongruent, and neutral RTs at the 0 ms SOA for both the attend auditory (auditory alone RT = 631.9 ms) and attend visual (visual alone RT = 570.5 ms) conditions (see Figure 2, top). The mean auditory alone RT was significantly slower than the congruent RTs (*M = *609.6 ms; *t*(14) = 2.26, *p = *0.04), but significantly faster than both the neutral (*M = *663.2 ms; *t*(14) = 3.00, *p = *0.01) and incongruent conditions (*M = *677.5 ms; *t*(14) = 3.96, *p = *0.001). The visual alone RT trended toward being faster than the congruent RT (*M = *586.8 ms; *t*(14) = 1.93, *p = *0.07), but was significantly faster than the incongruent (*M = *598.4 ms; *t*(14) = 3.09, *p = *0.008) and neutral RTs (*M = *599.8 ms; *t*(14) = 3.20, *p = *0.006). Additionally, a comparison between the two unimodal stimuli (auditory vs. visual) further confirmed that responses to the visual stimuli were significantly faster than responses to the auditory (*M* visual* = *573.9 ms; *M* auditory* = *633.8 ms; *t*(14) = 4.61, *p*<0.001).

### Normalized Data

**Figure 3:**
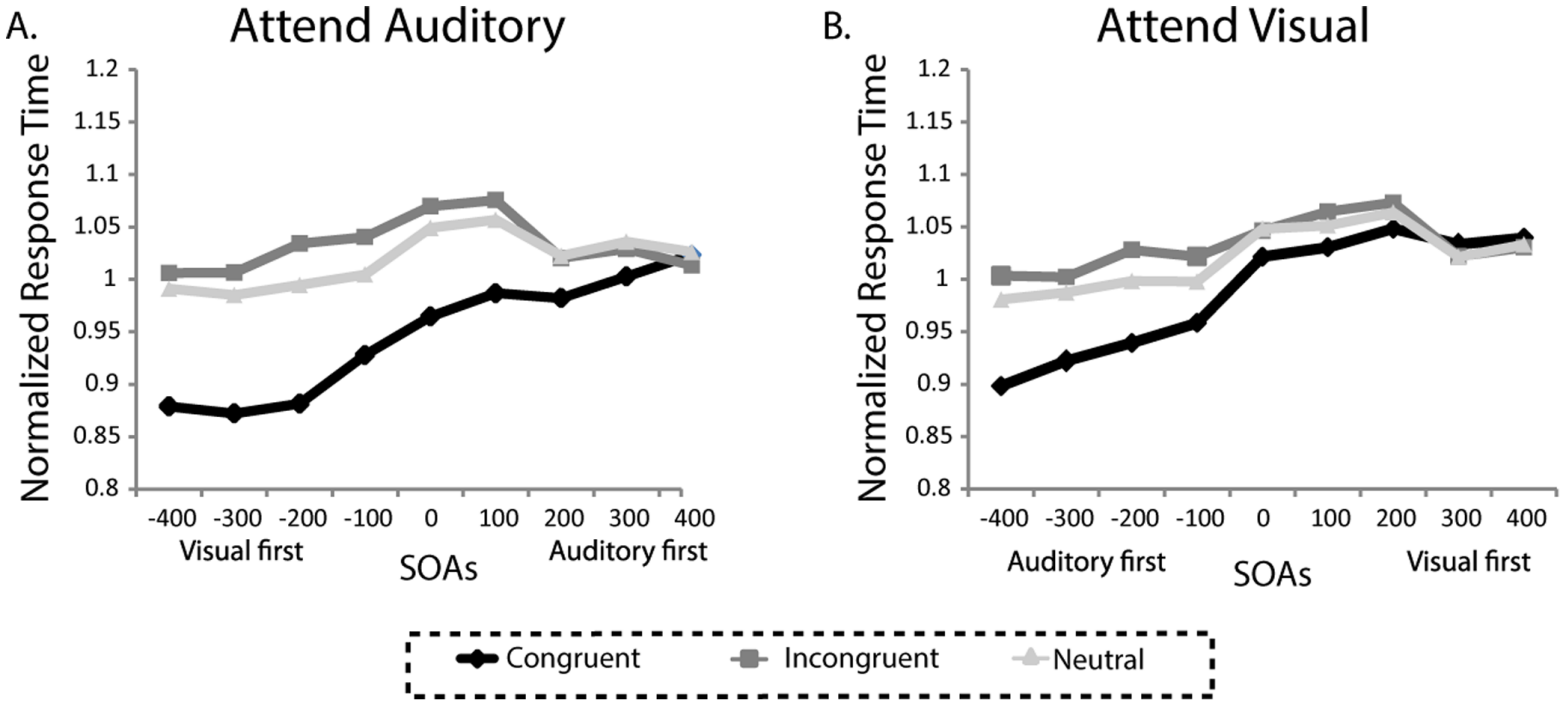
Normalized Response times. A. Normalized RTs for the attend auditory condition plotted as a function of SOA and congruency. A value of 1 means that the RT was the same as the unimodal control (auditory alone). B. Normalized RTs for the attend visual condition plotted as a function of SOA and congruency. A value of 1 means that the RT was the same as the unimodal control (visual alone).

Although the general pattern of results showed that the irrelevant visual stimuli had more of an influence on the processing of auditory stimuli, the auditory stimuli were also processed more slowly than the visual stimuli and as such any increased facilitation/interference effects may be mainly resulting from these baseline differences in processing speeds between the modalities. To adjust for the contribution of these baseline differences in processing speeds on the incongruency effects, we normalized the data (see Methods for in-depth description and Figure 3 for plots) and re-computed the same ANOVAs as above. This analysis revealed that there was still a main effect of SOA (*F*(8,112) = 33.80, *p*<0.001; η*_p_^2^* = .71), a main effect of incongruency (*F*(2,28) = 130.06, *p*<0.001; η*_p_^2^* = .90), an interaction of attended modality and incongruency (*F*(2,28) = 21.64, *p*<0.001; η*_p_^2^* = .61), and an interaction of SOA and incongruency (*F*(16,224) = 17.13, *p*<0.001; η*_p_^2^* = .55). There were no main effects of attended modality (confirming the normalization) and no significant interactions between any of the other factors (all *p*’s>0.05). Further examination of the modality by incongruency effect revealed that the full interference effects (incongruent vs. congruent) and facilitation effects (neutral vs. congruent) were greater when the auditory modality was attended than when the visual modality was attended (full incongruency: *t*(14) = 4.64, *p*<0.001; facilitation: *t*(14) = 6.54, *p*<0.001). Together, these data indicate that while attending to and discriminating the auditory stimulus components slowed RTs generally, the asymmetric pattern of facilitation and incongruency effects observed do not simply reflect differences in the baseline processing speeds for the two modalities.

### Psychometric Modeling

**Figure 4:**
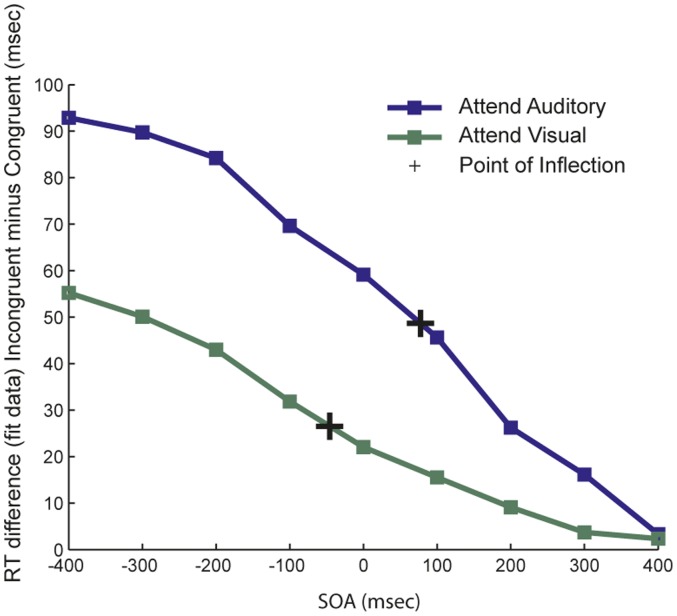
Average model fits. Plot of modeled data for attend auditory and attend visual conditions. The y-values represent the RT difference for mean model fits for incongruent trials minus the mean model fits for congruent trials and the x-values are the SOAs. The “+” signs represent the inflection points as determined by the fits for the corresponding conditions, with the inflection point for the ‘attend auditory’ condition occurring at a more positive (later) SOA than for the attend visual condition.

The best fitting sigmoidal functions were derived for each participant’s SOA by incongruency RT data, after collapsing over the random- and blocked-SOA arrangements in the attend auditory and attend visual conditions, as noted above. Figure 4 shows the differences between the mean congruent and incongruent model fits as a function of SOA for the attend visual (green) and attend auditory (blue) conditions. The respective inflection points for each function are marked with an “+.” If, indeed, interference processing lasted longer (i.e. spanned across more SOAs) for one attended modality than the other, we would expect to observe a rightward shift in the inflection point when that modality was attended. Such a shift would indicate interference lasting over more SOAs, implying that the processing time of these interference effects lasts over a greater time range. Across all subjects, the mean point-of-inflection was −45.8 ms for the visual fits, and 77.4 ms for the auditory fits. These values differed significantly [t(14) = 2.11, p = 0.05], indicating that visual distractors are processed faster and produce longer lasting interference, relative to auditory distractors on visual targets. The difference between the curve fitting estimates for the two modalities however, was not significantly different than the difference between the unimodal auditory and unimodal visual stimuli (curve shift = 123.2 ms, unimodal shift = 59.5 ms; (t(14) = 0.99, *p* = .34). This lack of interaction between incongruency and the attended modality suggests that the observed difference in conflict processing for the two modalities here did not significantly exceed the basic processing asymmetry for either stimulus type alone.

## Discussion

A rapidly expanding body of empirical research has been directed toward addressing how information presented to different senses is integrated and how incompatibilities between cross-modal stimuli are resolved to enable successful goal oriented behavior (for reviews see [31], [32]). The current study sought to delineate the time course of cross-modal interference in an audio-visual Stroop conflict paradigm. To this end, participants performed a modified version of the Stroop task in which they were instructed to attend to either the auditory or visual modality, and to ignore the irrelevant information in the other modality. The irrelevant information could be congruent, incongruent, or neutral with the attended-modality target and was presented across nine SOAs from −400 to +400 ms (relative to the relevant component), in both random and blocked SOA arrangements (separate sessions). Several major patterns of results emerged in the reaction time data obtained from this paradigm. First, visual distractors produced larger incongruency effects on auditory targets than vice versa at SOAs beyond just the simultaneous effects that had been previously reported (e.g., [10]). This was observed in both the raw and the modality-normalized data. Second, the processing of the visual information was faster than the auditory across multiple SOAs, and when vision was the irrelevant modality it also produced stronger and longer lasting incongruency effects (i.e., interference across more SOAs) on the auditory processing than the auditory distractors produced on the visual targets. This is evident in the overall faster RTs for visual than auditory targets, as well as through the shift in the psychometric interference functions where visual distractors interacted with auditory targets earlier (i.e. at more negative SOAs) and at a broader range of SOAs than vice versa. Third, as has been previously shown within the visual modality (e.g. [23], [25]), the cross-modal stimulus congruency relationship interacted with SOA, producing the larger behavioral effects when the irrelevant-modality distractor was presented earlier in time relative to the attended target. Fourth, and in contrast to intramodal visual conflict paradigms, these audio-visual stimuli resulted in substantially more behavioral facilitation than incongruency led to behavioral interference (relative to neutral stimuli as baseline). Finally, there were no main effects or interactions due to the arrangement of SOAs over trials, in contrast to what our group had previously shown for the visual modality. Interestingly, error rates were higher for the incongruent trails, but no other factor influenced accuracy in this task, suggesting that cross-modal conflict primarily alters the behavioral response speeds. Further discussion of these various RT effects follows.

### Asymmetric Magnitude of Incongruency Effects between Modalities

By using physically identical stimuli and manipulating attention to be directed toward either one sensory modality or the other, we observed larger incongruency effects from the visual distractors on the auditory processing, as compared to vice versa. Specifically, the overall incongruency effects, even when the data were modality-normalized, were still greater when participants were attending to the auditory modality than when they were attending to the visual modality. In general, this asymmetric pattern of the cross-modal incongruency effect, which occurred across the varied SOAs employed here, is in agreement with other studies, wherein the incongruency was in the form of multisensory objects using simultaneous audio and visual presentation (e.g., a dog saying “meow”, [10], [33]). However, an inference that can be made across these studies is that this modality dominance is not rigid. Rather, in terms of guiding behavior, the relative contribution of each modality seems to be influenced by the quality of the information that tends to be available from that modality, which may in turn make it more or less difficult to attend to and act upon that which is task-relevant (e.g., [34]). For spatial localization purposes, for example, when the naturally higher spatial resolution of visual stimulation is particularly informative, visual input affects auditory perception of location more than auditory stimulation affects that of the visual perception (e.g. as in the ventriloquism effect, where the perceived spatial location of an auditory stimulus is shifted toward that of a simultaneous visual input (e.g., [14])). In contrast, when the auditory information is particularly informative, such as for timing purposes, the relative importance of the modalities can switch (e.g., the sound-induced extra flash illusion wherein brief illusory flashes are perceived in response to the simultaneous occurrence of brief auditory tones [35]).

Our data therefore suggest that, in the specific case of written and spoken words, the brain accepts visual input as being more reliable than the auditory input and thus more heavily weights the visual input such that it exerts more of an influence on processing. This is somewhat surprising given that the visual stimuli were presented slightly parafoveally (3.75° below fixation) and that the auditory stimuli were presented at a relatively high volume, making them fairly hard to ignore. Further, the auditory stimuli were presented bilaterally through stereo speakers (located to the left and right of the monitor and hidden from view) and thus were perceived as coming from the same central location as the visual stimuli. Nevertheless, under the current experimental context it appears that visual information was more heavily weighted in the preparation and execution of the behavioral responses. It is also likely that had we substantially degraded the visual input as was done by [10], the greater reliance on the visual input would likely have been diminished. Future work could involve parametrically degrading the visual and auditory inputs to determine the relative degradation needed before one modality becomes more reliable than the other.

### The Asymmetric Timing of Modality-Specific Processing

A fundamental question addressed in the present experiments concerns the relative timing of visual and auditory information processing, and how potential modality differences may interact with the compatibility of the stimuli. For this purpose, we exploited the SOA-varying technique to investigate how temporal processing properties determine the time course of cross-modal behavioral incongruency effects. In line with previous reports using simultaneous audiovisual presentation (e.g. [9], [36], [37]) we observed that visual targets produced significantly faster RTs than auditory targets. In addition, however, we also observed in the present study a rightward shift in the interference psychometric functions, indicating that visual distractors interacted over a greater range of SOAs on the auditory targets, as compared to the converse. These data thus suggest that, because the visual distractors were more rapidly processed than the auditory distractors, they were able to interfere significantly even when they were presented up to several hundred milliseconds after the auditory stimuli.

By fitting these interference functions, we were able to delineate the temporal pattern of audio-visual interference, highlighting the relative discrepancies between the temporal processing of stimuli across modalities and how this ramifies into cross-modal processing interactions. While the difference in latencies of the sigmoidal psychometric functions (the rightward shift) did not statistically exceed that of the unimodal RT differences, they were in absolute terms roughly 60 ms larger (123 ms versus 60 ms), suggesting that there may be additional processing costs associated with cross-modal stimuli that we were unable to fully assess here. Given that reaction times represent the end point of a cascade of modal and amodal conflict interaction and resolution processes, it remains an open question as to exactly how these stimuli progress through the underlying neuro-cognitive architecture. By using neural measures with high temporal resolution, such EEG or MEG, future studies may be able to tease apart the temporal dynamics of, and thereby further inform us about, the relative speed versus relative strength of processing that underlies the conflict processing interaction across the different modalities.

It is also worth noting that some aspects of the temporal patterns observed here likely resulted from the specific types of relatively complex, linguistic stimuli used. Indeed, when simple auditory tones and visual flashes are presented, a different pattern of effects tends to be observed, with generally faster RTs for auditory targets than visual ones [38]. This difference however, is likely to be due at least in part, to the fact that the entire visual stimulus for word stimuli, such as we used here, is disclosed instantly at the start of the trial, whereas the corresponding auditory stimuli necessarily unfold over some time as the words are articulated. Such differences in the exposure of the stimuli are particularly important to consider in the context of the present design because the SOA approach offers a specific means by which to assess the relative timing of modal stimulus processing. Accordingly, in order to fully assess what the relative unimodal processing differences were, we obtained data with each of these stimuli presented alone, without any other distracting information, and we used these values to modality-normalize our data. The faster RTs for the visual unimodal control stimuli than for the auditory ones confirmed that in our case visual stimuli were indeed processed more rapidly than auditory. Importantly, however, because the current design used such a broad range of SOAs we were able to determine the relative timing of processing for the auditory and visual stimuli that remained constant across the different SOAs, thereby accounting for differences in the physical properties of the stimuli.

### Interactions between Congruency and SOA: Priming, Backward Influences, and General Alerting

Based on previous literature, it is well appreciated that the temporal relationship between the occurrences of near-synchronous stimuli greatly influences the processing and perception of those stimuli. One modulatory effect that has been well-characterized is priming, wherein a previously-presented stimulus can influence the perceptual processing of a subsequent target stimulus, leading to changes in response time or accuracy [39]. Priming generally manifests as a benefit in response time, with faster response times to targets that have been ‘primed’; however, priming can also serve to distract, producing negative or inhibitory influences on the target (e.g., [40]). It is also the case, however, that when irrelevant stimulus input follows a target in fairly close temporal proximity, it can have “backward” influences on the processing of the target (see [23]). That is, facilitation and interference can still be induced on the processing of the target due to the mere occurrence of a subsequent stimulus and its particular properties relative to the target (see [41]). Both priming and backwards interference have been demonstrated in a visual-tactile detection task, wherein congruent visual stimuli primed a correct tactile response and incongruent visual distractor stimuli produced interference, both before and after the target stimulus (for SOAs up to 100 ms; [42] see also [43] for another example of visual-tactile priming).

Results from the present study demonstrate that cross-modal stimuli evoke characteristics of both priming and backward influences. As observed in our previous unimodal visual studies [23], [24], we found here that incongruency interacted with SOA, with the largest incongruency effects (incongruent versus congruent conditions) occurring when the irrelevant dimension was presented first, and reduced but detectable effects when it was presented after. Here, however, this priming was predominantly driven by a significant speeding of RTs for the congruent stimuli (relative to neutral) when they occurred successively earlier, an effect that likely resulted from the redundancy of the congruent representation between the two modalities. Overall, these effects showed a monotonically reducing impact with later SOAs that was observed for both attended modalities (see Figures 2C and 2D) and therefore reveals a consistent pattern of interactions between SOA and the relative cross-modal stimulus congruency. It is likely, however, that priming is not the only process that has a facilitating influence on the negative SOA conditions. Across all trial types, having the irrelevant information appear first may have also served to alert the participants of an upcoming target, regardless of whether this information was congruent or incongruent with the target stimulus. Thus, the presentation of another stimulus prior to the target may have facilitated processing of the target in all of the negative SOA conditions, thereby speeding the RTs, which in turn may have resulted in slightly reduced interference effects observed during this time period.

Results from the neutral condition, are in fact, consistent with this possibility. Specifically a main effect of SOA was present, with the slope of this function indicating a general facilitation (lower RTs) at earlier SOAs. This result thus supports the inference that pre-exposure of task-irrelevant stimuli is to some degree serving to exert a general alerting influencing that affects performance on all negative-SOA trials, irrespective of the relative congruency with the attended target. While these behavioral results help disentangle the contribution of general alerting influences from facilitation and interference, the data pattern indicate that there is also greater congruency-related priming at the pretarget SOAs, as noted above, that is superposed on the general alerting effect.

### Facilitation, Interference and the Varying Forms of Stimulus Neutrality

Another pattern of results that emerged from these data is that cross-modal incongruency evoked substantially more behavioral facilitation than interference. This is particularly evident in the irrelevant-first SOAs where the congruent condition differed markedly from the neutral condition, while the incongruent condition only differed slightly. This observation, however, brings up an important question about the nature of “neutrality” in a cross-modal conflict task. Here, if we compare our congruent and incongruent RTs to the unimodal stimuli (“x” in Figures 2A and 2B), it is evident that this would give a vastly different pattern of results than comparisons with the neutral stimulus (lightest grey lines in Figures 2A and 2B). RTs to the unimodal stimuli fell far closer to the congruent stimuli, suggesting that there would be far more interference than facilitation present if these unimodal stimuli were taken as the neutral point of reference. In fact, a recent empirical and theoretical paper has shed important light on the types of interrelations that may be driving such RT differences [44]. As described by Brown, the interpretation of the Stroop task must distinguish between nonspecific lexicality-based effects and specific color-word congruency effects. That is, a word that is known (i.e., here, every one of the color words, including the “neutral” ones) has a meaning, and this meaning (or lexicality) has an influence on the processing of these stimuli apart from whether the meaning is directly congruent or incongruent with the desired response. In particular, when compared to regularly-spelled but meaningless non-words, meaningful words require greater processing times due to the activation of lexical representation of these stimuli. It is this “lexicality cost” that Brown argues has confounded much of the existing interpretation of the Stroop behavioral literature.

In the current design the neutral stimuli have just this type of lexical relationship, and therefore such lexicality effects may have indeed interacted with the overall dynamics captured in the present findings. That is, instead of using neutral stimuli that were non-words (e.g., the letters “XXXXX” or auditory noise), our neutral stimuli were words, and therefore contained to-be-processed meaning. Importantly, however the unimodal stimuli in our design offer a principled reference point that supports the notion of a lexical cost for the neutral stimuli (albeit necessarily just for the 0 ms SOA condition). Whereas the neutral stimuli differ from congruent and incongruent stimuli, both in terms of lexicality and congruency, the unimodal stimuli have no other sensory competition. Therefore, the difference between the unimodal stimuli and the neutral stimuli (with the neutral stimuli producing somewhat slower RTs) can be viewed as the extra lexical cost associated with the processing of these meaningful stimuli arising from a different modality. Potential distraction by this lexicality for both the neutral and incongruent stimuli could thus explain why the alerting effect for these stimuli on the eventual RTs was relatively less than for the congruent stimuli. In addition, while incongruency adds additional behavioral slowing, the substantial difference between the neutral condition and the congruent and unimodal controls suggests that the lexical processing of these neutral stimuli was the dominant factor diverting attention away from the relevant modality. It is worth noting however, that this pattern differs somewhat from our previous visual Stroop-SOA task [23], where we observed that RTs to the neutral stimuli fell rather evenly between the congruent and incongruent RTs, leading to proportionally less facilitation in contrast to what we observed here. As the putative degree of lexicality should be the same for the stimuli in the two experiments, yet additional factors may be influencing the perception of temporally separated cross-modal stimulation. Future research will be needed to disentangle how these factors are differentially expressed in unimodal and cross-modal situations.

Although our results indicate the presence of substantial amounts of facilitation, one previous cross-modal Stroop SOA conflict study by Roelofs [5] did not observe such a pattern, reporting instead more interference than facilitation when participants were instructed to name visual color patches, or visual words, while ignoring spoken words. This discrepancy is likely based, at least in part, by the fact that the reference point that Roelofs used for computing facilitation and interference was in relation to a unimodal stimulus, rather than a visual color stimulus that was not mapped to any response, as was the case with our neutral stimuli. When taking this difference into consideration, Roelofs’ unimodal stimuli and ours generally fell close to the congruent RTs, which would thereby produce more interference if this was the relative comparison. It is also worth noting, however, that our pattern of results differed in another, perhaps more substantial, way from those of Roelfs. Namely, we observed greater interference from irrelevant-visual stimuli on relevant-auditory stimuli, where as he found the opposite pattern. One possible explanation for this difference could be that the present study required subject to respond manually, while the Roelofs’ study required verbal responses. As noted by others (e.g., [16]) the manifestation of stimulus-response conflict depends on the degree to which the stimulus representation needs to be translated in order to map onto the various response options. Accordingly, this difference in response mode may account for the overall difference in the pattern of congruency effects for these two tasks.

### Stimulus vs. Response Conflict

Thus far we have described the relative facilitation versus interference effects observed here within the context of lexicality costs. Nonetheless another important dimension that is worth noting is the underlying locus of these incongruency effects. Incongruency can come at the level of the stimulus (e.g., where the word “Red” is written in blue ink such as in a typical Stroop task), or at the level of the response (e.g., where there are multiple response options for a given input). Each of these factors can lead to slowed RTs, but they may act to do this via different mechanisms. Specifically, when multiple responses compete for representation, the anterior cingulate cortex (ACC) would appear to be involved in this competition, wheres it may not be at the level of stimulus conflict [45].

In the current study, both our neutral stimuli and our incongruent stimuli did not match the to-be-attended stimulus in meaning, and were therefore semantically incongruent; however, there were still differences in RTs between the neutral and incongruent stimuli, particularly observed for the negative SOAs. These differences, therefore, are likely attributable to response conflict that was present in the incongruent stimuli but not present in the neutral stimuli as the neutral stimuli were not mapped to a response. Many studies have examined the differences between stimulus and response conflict (e.g., [46], [47], [48]) with response conflict generally adding interference above and beyond what is present for stimulus conflict. Indeed, having response conflict on top of stimulus conflict here further slowed RTs suggesting that interference was occurring at both the stimulus and response level.

### Timing Context

The final, and perhaps somewhat surprising, finding from the present study was the lack of a main effect or interaction as a function of the SOA arrangements. Previous work from our lab using variants of the visual Stroop task [24] found that when the SOAs were blocked, such that the same SOA appears across all trials in an experimental run, the pattern of behavioral and neural incongruency effects appeared as an inverted U function. That is, the greatest incongruency effects occurred with simultaneous presentations (0 ms SOAs) and decreased monotonically in either direction, as the relative SOAs got larger. In contrast, when the SOAs were presented in a random order, a strong pattern of incongruency-related priming was evident, with the greatest effects coming at the earliest pre-exposure SOAs and decreasing as the incongruent distractor occurred later. These within-modality results thus suggested that participants are able to set up a temporally-based attentional filter in order to mitigate the influence of incongruency when the temporal arrangement of the stimulation is predictable.

The present cross-modal results, showing the priming pattern for both SOA configurations, indicate that this differential pattern as a function of SOA arrangement present within the visual modality does not extend to cross-modal stimuli. As shown in previous temporal judgment experiments (e.g., [49]), it is relatively easy to distinguish the relative separation of two visual stimulus components once they differ by more than 50 ms. In the case of cross-modal processing, temporal-order judgments are not so precise and there is much more room for temporally discrepant auditory and visual stimuli to be perceived as simultaneous. In fact, in most cross-modal simultaneity judgment tasks, the stimuli need to be more than 150 ms apart for participants to begin to reliably distinguish them and up to 300 ms or more before participants consistently distinguish them in time [50], [51], [52]. We infer, therefore, that in the current cross-modal experiment the stimuli were simply too close in time for participants to be able to detect a reliable temporal pattern and modulate their behavior accordingly.

On the other hand, it is possible that there simply was not a wide enough range of SOAs tested to obtain such a main effect of SOA arrangement. To examine for this possibility post-hoc, we took the −400 condition alone and conducted an ANOVA to determine if this SOA, which should be well outside the temporal window of integration, would show differential congruency effects as a function of the SOA arrangement. Although there were no main effects of SOA arrangement, or interactions between SOA arrangement and congruency when the auditory stimuli were attended, when the visual stimuli were attended there was indeed a main effect of SOA arrangement *F*(1,14) = 15.03, *p = *0.002), as well as an interaction between SOA arrangement and congruency (*F*(2,28) = 27.68, *p*<0.001). More specifically, when the SOA arrangement was mixed (i.e., unpredictable) there was a bigger difference between the congruent and incongruent stimuli (77 vs 63 ms), which implies that when the stimuli were blocked participants may have been better able to filter out the irrelevant stimuli. This suggests that extending the audio-visual SOAs to great asynchronies could possibly reveal a similar pattern to that we found previously with the visual stimuli; on the other hand, such a manipulation may not be practical because at such extreme separations congruency interactions may not occur. Nevertheless, this pattern of results is in line with previous work highlighting the broad temporal window of integration observed for cross-modal stimulus processing (e.g., [53]).

### Temporal and Semantic Forms of Cross-modal Conflict

In the present design there are a number of different dimension under which the cross-modal stimuli may correspond or conflict. Thus far we have discussed both the semantic/lexical relationships between these stimuli, as well as the degree to which these may engender correspondence or conflict at the level of sensory or response processing. The SOAs used to separate these stimuli, however, also may lead to differences in the temporal binding of the two modalities. Namely, since the more extreme positive and negative SOAs fall outside of the temporal window of integration [29], [54] under which auditory and visual stimuli may bind into a single perceptual object, this may lead to differing amounts of temporal conflict.

Recently, there has been some interesting work examining the effect of conflict on temporal perception, using tasks that tap into effects of temporal integration of cross-modal stimuli (see [55]). Specifically, when participants are asked to make judgments about the timing of semantically congruent or incongruent speech stimuli, participants are more likely to temporally integrate the audio-visual information if they are congruent as compared to incongruent [56], [57]. That is, the congruency of the information broadens the window over which the stimuli will be related.

In the present study we do not have a direct measurement of temporal integration, yet it is still likely that some sensory integration was occurring here. First, examining the congruent condition alone, it is very likely that multisensory integration is occurring across the majority of SOAs. This is due to both the semantic matching as well as the spatial and temporal overlap of the audio-visual stimuli. However, at the largest SOAs within the congruent condition, there is likely temporal conflict (i.e., +/−400 ms) as these are outside the temporal window of integration, and under such circumstances the stimuli are likely no longer integrated. Nevertheless, we still see some behavioral effects of facilitation (priming) at the −400 ms SOA, suggesting that although multisensory integration is likely not due to the vast temporal separation, there is still some response-facilitation due to overlap in stimulus-response mapping. In the case of the neutral and incongruent conditions, the level of multisensory integration occurring here is probably only occurring at a relatively low processing level based on the temporal and spatial overlap of the stimuli. Together, all of these conditions manipulate integration to varying degrees while pitting together two competing sensory inputs (e.g., [58]). It would be of great interest to have neural measures to give additional insight into the degree to which all of these factors interact and when sensory integration is occurring with these stimuli.

### Conclusions

In sum, the present data demonstrate that irrelevant visual information affects the processing of relevant auditory information more than vice versa, and it does so over a broad time-occurrence scale. These patterns of behavioral effects are largely dominated by RT facilitation, although differences between performance for unimodal and neutral control stimuli suggest that lexicality effects may be influencing these interactions. Finally, unlike in our previous visual Stroop tasks, there was no influence of the SOA arrangement, possibly due to the broad temporal window over which cross-modal stimuli tend to interact and be perceived as occurring simultaneously. Overall, the pattern of effects reported here highlight key differences between cross-modal and within-modality conflict processing. Indeed, these differences would appear to be critical to consider when studying cross-modal information processing.
